# Thromboelastography Associates to Decreased Plasma Transfusions in the Medical Intensive Care Unit: A Retrospective Study

**DOI:** 10.1155/ccrp/5575261

**Published:** 2026-04-21

**Authors:** Daniel F. Lewandowski, Connor M. Bunch, Morgan N. Howard, Chun-Hui Lin, Fiona G. Clowney, Austin J. Parsons, Michael J. Sheehan, Sulmaz Zahedi, Kristopher Mosier, Rachel A. Eklund, Ileana Lopez-Plaza, Joseph B. Miller, Jennifer L. Swiderek

**Affiliations:** ^1^ Department of Pulmonary and Critical Care Medicine, Henry Ford Hospital, Detroit, Michigan, USA, henryford.com; ^2^ Department of Emergency Medicine, Henry Ford Hospital, Detroit, Michigan, USA, henryford.com; ^3^ Department of Internal Medicine, Henry Ford Hospital, Detroit, Michigan, USA, henryford.com; ^4^ Department of Pulmonary and Critical Care Medicine, Trinity Health Ann Arbor Hospital, Ann Arbor, Michigan, USA; ^5^ Department of Public Health Sciences, Henry Ford Hospital, Detroit, Michigan, USA, henryford.com; ^6^ Department of Transfusion Medicine, Henry Ford Hospital, Detroit, Michigan, USA, henryford.com; ^7^ Department of Emergency Medicine, Michigan State University Health Sciences, East Lansing, Michigan, USA

**Keywords:** blood coagulation disorder, blood transfusion, critical illness, resuscitation, thromboelastography

## Abstract

Bleeding patients in the medical intensive care unit (MICU) are often coagulopathic, yet the best way to guide blood product resuscitation for the critically ill is not settled. Viscoelastic tests, such as thromboelastography (TEG), are increasingly used by intensivists to guide resuscitation and restore hemostasis. We performed a retrospective study of patients admitted to the MICU at a single tertiary care center in Detroit, Michigan, USA, with a high prevalence of decompensated cirrhosis and septic shock. The historical cohort included patients prior to TEG being available in the MICU. The observational cohort included data where TEG was available and applied in our MICU to guide blood product use. Patients were included if they were an adult > 18 years of age with an initial admission to Henry Ford Hospital MICU who received any blood component therapy. A total of 927 patients met inclusion criteria with 487 (52.5%) patients in the historical cohort and 440 (47.5%) in the observational cohort. Compared with the historical cohort, patients of the observational cohort were administered significantly less plasma (658.3 mL/patient (IQR = 931) vs. 410.8 mL/patient (IQR = 381), *p* < 0.001). There was no significant difference in mortality (54.4% vs. 50.5%, *p* = 0.408) or hospital length of stay (LOS) (13 days vs. 14 days, *p* = 0.08). In a generalized MICU population, the introduction of TEG‐guided blood component resuscitation was associated with conserved plasma without significant effect on mortality or LOS. No causation can be drawn to TEG from the retrospective nature of this study. However, this study provides impetus to further study blood product stewardship potentially derived from viscoelastic test‐guided transfusions in a general MICU population.

## 1. Introduction

Critically ill patients often have disordered hemostasis due to a wide variety of disease processes and trauma [1]. For the bleeding patient, prompt recognition and treatment of coagulopathy are crucial for preventing the progression of hemorrhagic shock to organ dysfunction and death. Yet, blood products for treating the hemorrhaging patient such as packed red blood cells (PRBC), platelets, plasma, and cryoprecipitate are precious resources to be adjudicated carefully. Viscoelastic tests (e.g., thromboelastography [TEG]) are rapid bedside tools that help guide hemostatic therapy in hemorrhaging patients [2, 3]. TEG assesses clot formation on whole blood, from clot initiation and amplification to the maximal platelet–fibrin contraction strength and ultimately clot dissolution via fibrinolysis thus allowing clinicians to individualize therapy. TEG accurately and globally reflects the changes in patients’ hemostasis as seen in disease states such as decompensated cirrhosis with large variceal bleeds, trauma‐induced coagulopathy, and sepsis‐induced coagulopathy [4].

Prior studies on TEG‐guided blood component therapy have demonstrated reduced blood product use when compared with conventional coagulation tests (i.e., activated partial thromboplastin time [aPTT], prothrombin time [PT], and international normalized ratio [INR]) in guiding the resuscitation of coagulopathic patients such as those with liver failure, liver transplantation, trauma‐induced coagulopathy, postpartum hemorrhage, and cardiac surgery [1, 5–7]. The goal of this study was to add to the growing literature on TEG‐guided resuscitation, and to determine the effect of TEG‐guided coagulopathy correction on a general medical intensive care unit (MICU) population when compared with conventional coagulation studies on blood product consumption. While intensivists have been using TEG for years to help guide transfusion in episodes of acute bleeding, there are few studies focused specifically on a general MICU population, which at our center has a high prevalence of cirrhotic patients and septic shock. Further subanalysis was conducted to determine if there was a relation between 30‐day mortality and hospital length of stay (LOS). This article was written following the guidelines of the Strengthening the Reporting of Observational Studies in Epidemiology (STROBE) statement [8].

## 2. Methods

We performed an observational cohort study of patients admitted to the MICU at a single tertiary care center in Detroit, Michigan, USA. This study was approved under the Henry Ford Health System institutional review board and conducted in accordance with the Declaration of Helsinki. Henry Ford Hospital is a tertiary referral center which receives transfers throughout the state of Michigan for subspecialty services and particularly for transplant evaluations. Henry Ford Hospital provides the largest number of liver transplants in the state of Michigan annually and thus has a high census of decompensated cirrhotic patients.

**FIGURE 1:**
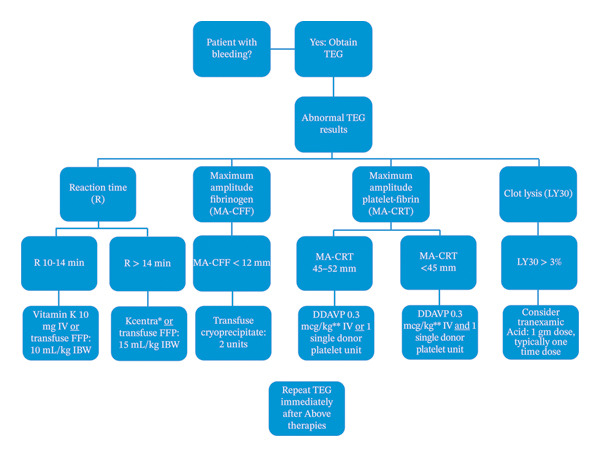
TEG‐based protocol for coagulopathy reversal at Henry Ford Hospital. ^∗^Consult pharmacy for one‐time Kcentra dosing. Kcentra is not indicated for repeat dosing. ^∗∗^Actual body weight, no max dose. Abbreviations: CFF, citrated functional fibrinogen; CRT, citrated RapidTEG; DDAVP, desmopressin; FFP, fresh frozen plasma; IBW, ideal body weight; IV, intravenous; LY30, lysis at 30 min; MA, maximum amplitude; R, reaction time; TEG, thromboelastography.

The historical cohort included patients from July 2019 to December 2019 prior to TEG being available in the MICU. The observational cohort included data collected from July 2023 to December 2023 where TEG was available and applied in our MICU to guide blood product use. No effort was made to restrict the use of conventional coagulation studies. A supplemental protocol on TEG‐guided coagulopathy correction was posted in the clinicians’ offices (Figure 1). Prior to introduction of TEG, a large formal lecture was provided to residents, fellows, attendings, midlevel providers, and nurses. Additionally, since the introduction of the TEG to Henry Ford Hospital, monthly TEG lectures are given to the rounding residents. If massive transfusion protocol was activated or multiple blood component therapies were administered without apparent coagulopathy correction, our blood banking team mandated a TEG be collected, and subsequent transfusions thereafter would deviate from fixed ratio resuscitation to a TEG‐guided goal‐directed resuscitation in a collaboration between the blood banking team and primary ICU team.

We reviewed patients who met the following inclusion criteria: adults > 18 years of age with their first admission to Henry Ford Hospital MICU and who received any blood component therapy. Patients were excluded from the study if clinical data was incomplete (i.e., partial or duplicate charts) or components of their social or past medical history were missing. Please note that “unknown” alcohol use history documented in the chart was treated as a separate category, but these patients were not excluded on unknown alcohol use history alone so long as the remaining aspects of the chart were complete. Patients were excluded from the observational cohort if a TEG was not obtained by the clinical team. All TEGs were collected on the cartridge‐based TEG 6s device with either the Global Hemostasis or Global Hemostasis with Lysis cartridges (manufactured by Haemonetics in Boston, Massachusetts).

Data were collected via Clarity, a database from the Epic electronic medical record, using Microsoft SQL Server Management Studio. Comorbidities were obtained via their charted ICD‐10 codes, and transfusion data came from both documentation and transfusion orders placed by providers. TEG usage was compared via Wilcox rank‐sum tests for continuous variables and/or chi‐squared testing for categorical variables.

## 3. Results

**FIGURE 2:**
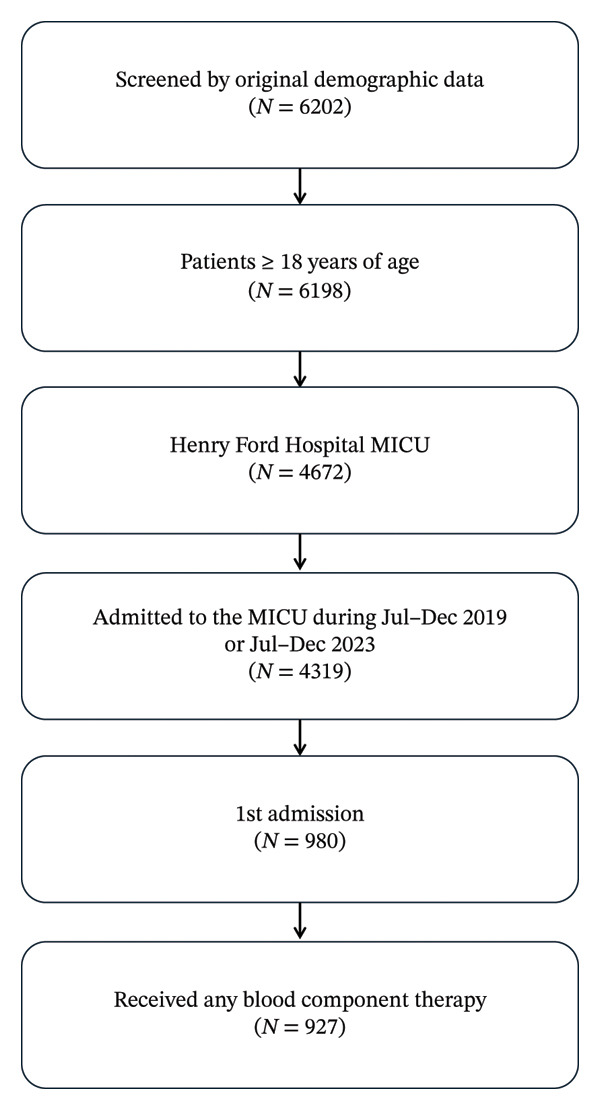
Study cohort flow diagram. Abbreviation: MICU, medical intensive care unit.

Of 6202 patients screened for the study, 927 patients met inclusion criteria with 487 (52.5%) patients in the historical cohort and 440 (47.5%) in the observational cohort (Figure 2). The average age was 58.8 years with 48% female and 52% male. The prevalence of alcohol use, body mass index, liver disease, liver failure, congestive heart failure, coronary artery disease, and diabetes were similar between the two groups. The Charlson Comorbidity Index (CCI) was applied to both groups to ensure similar amounts of equally comorbid patients in each group (Table 1).

**TABLE 1:** Patient characteristics.

	**Overall**	**2019: Historical (non-TEG)**	**2023: Observational (TEG)**	**p** **value**

Total, *n* (%)	927	487 (52.5)	440 (47.5)	
Age (years), median (IQR)	61.0 (48.5–70.0)	60.0 (48.0–70.0)	62.0 (49.0–70.3)	0.39
Gender, *n* (%)				0.534
Female	445 (48.0)	239 (49.1)	206 (46.8)	
Male	482 (52.0)	248 (50.9)	234 (53.2)	
BMI (kg/m^2^), median (IQR)	28.0 (23.7–33.6)	28.2 (23.6–34.2)	27.8 (23.9–33.3)	0.673
Alcohol use, *n* (%)				< 0.001
Current	196 (21.1)	113 (23.2)	83 (18.9)	
Past	271 (292)	127 (26.1)	144 (32.7)	
Never	302 (32.6)	189 (38.8)	113 (25.7)	
Unknown	158 (17.0)	58 (11.9)	100 (22.7)	
CCI, median (IQR)	3.0 (1.0–5.0)	3.0 (1.0–5.0)	3.0 (1.0–5.0)	0.361
0 – 2, *n* (%)	353 (38.1)	195 (40.0)	158 (35.9)	0.361
3 – 4, *n* (%)	300 (32.4)	149 (30.6)	151 (34.3)	
5+, *n* (%)	274 (29.6)	143 (29.4)	131 (29.8)	
Liver disease, *n* (%)	186 (20.1%)	98 (20.1%)	88 (20.0%)	0.712
CAD, *n* (%)	168 (18.1%)	84 (17.2%)	84 (19.1%)	0.521
Diabetes, *n* (%)	256 (27.6%)	139 (28.5%)	117 (26.6%)	0.555

*Note:* IQR, interquartile range.

Abbreviations: BMI = body mass index, CAD = coronary artery disease, CCI = Charleson Comorbidity Index.

**FIGURE 3:**
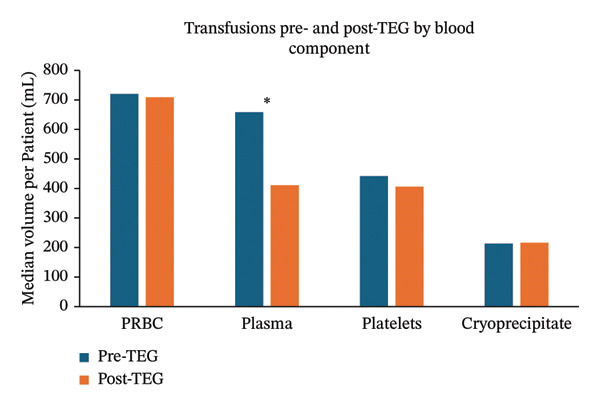
Median volume of blood product given to patients in the MICU. ^∗^ *p* < 0.001. Abbreviation: PRBC, packed red blood cells; TEG, thromboelastography.

Compared with the historical cohort, patients of the observational cohort were administered significantly less plasma (658.3 mL/patient (IQR = 931) vs. 410.8 mL/patient (IQR = 381), *p* < 0.001) as well as less PRBC (720.0 mL/patient (IQR = 1080) vs. 709.2 mL/patient (IQR = 755), *p* = 0.037) (Figure 3). While it did not reach significance, the patients in the observational cohort trended toward less platelet used while cryoprecipitate usage remained unchanged (Table 2). There was no significant difference in mortality (54.4% vs. 50.5%, *p* = 0.408) or hospital LOS (13 days vs. 14 days, *p* = 0.08) (Table 2).

**TABLE 2:** Blood component use, 30‐day mortality, and hospital length of stay in historical and observational cohorts.

	**Overall**	**2019: Historical (non-TEG)**	**2023: Observational (TEG)**	**p** **value**

Patients, *n* (%)	927	487 (52.5)	440 (47.5)	
Cryoprecipitate, median (IQR) in mL/patient	213.3 (111.0–323.0)	213.3 (110.5–318.5)	216.0 (112.1–329.8)	0.731
Plasma, median (IQR) in mL/patient	579.2 (325.0–1024.0)	658.3 (335.0–1265.7)	410.8 (300.0–680.7)	< 0.001
Platelets, median (IQR) in mL/patient	425.6 (233.0–769.5)	441.8 (256.2–753.5)	405.7 (218.4–768.6)	0.235
PRBC, median (IQR) in mL/patient	720.0 (360.0–1350.2)	720.0 (360.0–1440.0)	709.2 (360.0–1115.3)	0.037
Total cryoprecipitate (mL)	35,183	15,687	19,496	n/a
Total plasma (mL)	232,764	175,476	57,288	n/a
Total platelets (mL)	147,513	81,162	66,351	n/a
Total PRBC (mL)	894,337	480,100	414,237	n/a
Death, *n* (%)	482 (52%)	260 (53.4%)	222 (50.5%)	0.408
Hospital LOS, median (IQR) in days	13.0 (7.0–24.0)	13.0 (6.0–24.0)	14.0 (7.0–25.0)	0.08

*Note:* IQR, interquartile range; TEG, thromboelastography.

Abbreviation: pRBC = packed red blood cell.

## 4. Discussion

Our study demonstrated that the availability and use of TEG in a general MICU population was associated with decreased administration of plasma and PRBC. However, the marginal difference in median PRBC volumes may be a statistical phenomenon, which is not clinically significant. Moreover, TEG is not useful for directing PRBC transfusions in support of oxygen‐carrying capacity; rather, the TEG finds use in directing those blood components which may correct coagulopathies (i.e., plasma, platelets, and fibrinogen concentrates). There was a nonsignificant trend toward less platelet use and improved mortality. Conversely, there was a nonsignificant trend toward longer hospital LOS. Cryoprecipitate transfusion was similar pre‐ and post‐TEG implementation.

While this study demonstrates the application of TEG among a general MICU population, the first clinical application of TEG was for orthotopic liver transplantation. Physicians in the 1980s utilized TEG to improve hemostatic resuscitation during the vacillating coagulopathies and associated massive blood loss of liver transplantation [1]. The improvement in coagulation correction seen in liver transplantation paved the way for TEG to be applied to patients undergoing cardiopulmonary bypass surgery due to the need for balance between anticoagulation and hemostasis in the intraoperative and postoperative periods [5, 9]. In the late 1990s, traumatologists also began applying TEG‐guided massive transfusion for severely injured patients and has since demonstrated reduced blood product utilization and improved mortality in patients with trauma‐induced coagulopathy presenting to the emergency department, leading to its inclusion in the European guidelines in the management of severe trauma since 2013 [10–13]. TEG similarly assists heparin titration for patients on extracorporeal membrane oxygenation (ECMO) at some institutions [14, 15]. Most recently, TEG has been widely used in the MICU for treating the critically ill patient with COVID‐19 in a pandemic period bereft of evidence for anticoagulating patients who would often clot and bleed simultaneously [16].

Patients in the MICU have a wide range of pathologies, and TEG has been shown to accurately reflect the changes in patients’ hemostasis as seen during decompensated cirrhosis, acute illnesses, and infections [2]. Furthermore, it is common for patients with coagulopathies to require invasive procedures while in the MICU. A randomized controlled trial looking at patients with cirrhosis undergoing procedures showed significant improvement in the utilization of blood products by targeting TEG data rather than INR and platelet count with no increase in procedural complications [17]. Conversely, although shown in a general rather than cirrhotic population, a retrospective study showed increase in blood product utilization when using TEG to guide management over standard of care [18]. Like the INR and platelet count, derangements in TEG measurements correlate with cirrhosis severity and the model for end‐stage liver disease (MELD) score [19]. The most recent 2019 American Gastroenterology Association (AGA) practice update on coagulation monitoring in cirrhosis did not formally recommend viscoelastic test guidance but stated that these tests “may eventually have a role in the evaluation of clotting in patients with cirrhosis, but currently lack validated target levels” [20].

The results of our study agree with a prior systematic review including 1493 patients and a separate meta‐analysis of 1238 patients, with major bleeding demonstrating similarly decreased PRBC and plasma administration when guided by viscoelastic testing [21]. Additionally, smaller observational studies in patients with gastrointestinal bleeds, postpartum hemorrhage, and major trauma have shown increased cryoprecipitate and fibrinogen concentrate use with viscoelastic‐guided algorithms [22]. Provided that the predominant empiric hemorrhagic resuscitation strategy in the United States employs the 1:1:1 PRBC:plasma:platelets fixed ratio, without the inclusion of cryoprecipitate or fibrinogen concentrates, it is no surprise that deviation from the fixed ratio to a goal‐directed TEG‐guided strategy reduced plasma use [23]. The large discrepancy in the IQR between the historical and interventional cohorts in our study suggests that a relatively small subset of outliers in the historical group used significantly larger amounts of PRBC. Moreover, our study and the literature consistently show decreased plasma administration when guided by viscoelastic tests, as patients rarely demonstrate the need for plasma coagulation factor replenishment on viscoelastic tests except in the most severe cases of hemorrhagic blood failure [24].

The limitation of this study design is that it was retrospective, nonrandomized, and our intervention was primarily education on the use of TEG. As such, we did not restrict providers from using conventional coagulation tests or limit their bedside management of coagulopathy in any manner. Although the protocol was implemented throughout the MICU, participation was purely voluntary. We excluded from the interventional cohort patients who received blood products but in which a TEG was not ordered. We had no ability to isolate data on patients in the interventional cohort in which a TEG was ordered but the clinician utilized conventional coagulation factors and ignored the TEG to guide their blood product replacement. Furthermore, it was underpowered to validate the nonsignificant trends on platelet usage and 30‐day mortality. Moreover, given the observational nature and the possibility of confounding variables not accounted for (i.e., were clinicians guiding transfusions with the TEG or common coagulation tests), it is not possible to draw any causal relationship between TEG use and reduction in blood component use. The training and heightened awareness of blood product stewardship alone may have significantly contributed to the observed results. The reduced exposure to blood products may also have clinical significance independent of mortality or hospital LOS (e.g., reducing risk of circulatory overload, transfusion‐related lung injury, and infection complications); however, our study was not designed or powered to detect differences in these outcomes.

The strength of our design was that it was a real world, implementation study. When clinicians have access to both TEG and conventional coagulation tests, the implementation of a TEG‐based protocol resulted in a significant reduction in plasma use. Furthermore, the general MICU population consisted of patients with various pathologies as compared with the previous trials that focused on specific applications of TEG.

## 5. Conclusion

In a generalized MICU population, the implementation of a TEG‐guided blood component resuscitation protocol may conserve blood products—specifically plasma—without an effect on mortality. Further research with a rigorous prospective randomized comparison of conventional coagulation studies compared with TEG in a generalized MICU population is necessary to assess blood product conservation, outcomes data, and economic impact of TEG‐guided resuscitation.

## Funding

No funding was received for this manuscript.

## Conflicts of Interest

The authors declare no conflicts of interest.

## Data Availability

The data used to support the findings of this study are available on request from the corresponding author.

## References

[1] Kang Y. G. , Martin D. J. , Marquez J et al., Intraoperative Changes in Blood Coagulation and Thrombelastographic Monitoring in Liver Transplantation, Anesthesia & Analgesia. (1985) 64, no. 9, 888–896, 10.1213/00000539-198509000-00008. 3896028 PMC2979326

[2] Dias J. D. , Sauaia A. , Achneck H. E. , Hartmann J. , and Moore E. E. , Thromboelastography-Guided Therapy Improves Patient Blood Management and Certain Clinical Outcomes in Elective Cardiac and Liver Surgery and Emergency Resuscitation: A Systematic Review and Analysis, Journal of Thrombosis and Haemostasis. (2019) 17, no. 6, 984–994, 10.1111/jth.14447, 2-s2.0-85065726801. 30947389 PMC6852204

[3] Walsh M. , Fritz S. , Hake D et al., Targeted Thromboelastographic (TEG) Blood Component and Pharmacologic Hemostatic Therapy in Traumatic and Acquired Coagulopathy, Current Drug Targets. (2016) 17, no. 8, 954–970, 10.2174/1389450117666160310153211, 2-s2.0-84974663278. 26960340 PMC5374842

[4] Papatheodoridis G. V. , Patch D. , Webster G. J. , Brooker J. , Barnes E. , and Burroughs A. K. , Infection and Hemostasis in Decompensated Cirrhosis: a Prospective Study Using Thrombelastography, Hepatology. (1999) 29, no. 4, 1085–1090, 10.1002/hep.510290437, 2-s2.0-0032898078. 10094951

[5] Holcomb J. B. , Minei K. M. , Scerbo M. L. et al., Admission Rapid Thrombelastography Can Replace Conventional Coagulation Tests in the Emergency Department: Experience with 1974 Consecutive Trauma Patients, Annals of Surgery. (2012) 256, no. 3, 476–486, 10.1097/sla.0b013e3182658180, 2-s2.0-84865482138. 22868371

[6] Shore-Lesserson L. , Manspeizer H. E. , DePerio M. , Francis S. , Vela-Cantos F. , and Ergin M. A. , Thromboelastography-Guided Transfusion Algorithm Reduces Transfusions in Complex Cardiac Surgery, Anesthesia & Analgesia. (1999) 88, no. 2, 312–319, 10.1213/00000539-199902000-00016. 9972747

[7] Bugaev N. , Como J. J. , Golani G et al., Thromboelastography and Rotational Thromboelastometry in Bleeding Patients with Coagulopathy: Practice Management Guideline from the Eastern Association for the Surgery of Trauma, Journal of Trauma and Acute Care Surgery. (2020) 89, no. 6, 999–1017, 10.1097/ta.0000000000002944. 32941349

[8] von Elm E. , Altman D. G. , Egger M. , Pocock S. J. , Gotzsche P. C. , and Vandenbroucke J. P. , The Strengthening the Reporting of Observational Studies in Epidemiology (STROBE) Statement: Guidelines for Reporting Observational Studies, Annals of Internal Medicine. (2007) 147, no. 8, 573–577, 10.7326/0003-4819-147-8-200710160-00010, 2-s2.0-35848948166. 17938396

[9] Ganter M. T. and Hofer C. K. , Coagulation Monitoring: Current Techniques and Clinical Use of Viscoelastic point-of-care Coagulation Devices, Anesthesia & Analgesia. (2008) 106, no. 5, 1366–1375, 10.1213/ane.0b013e318168b367, 2-s2.0-42449112966. 18420846

[10] Gonzalez E. , Moore E. E. , Moore H. B et al., Goal-Directed Hemostatic Resuscitation of trauma-induced Coagulopathy: a Pragmatic Randomized Clinical Trial Comparing a Viscoelastic Assay to Conventional Coagulation Assays, Annals of Surgery. (2016) 263, no. 6, 1051–1059, 10.1097/sla.0000000000001608, 2-s2.0-84952674051. 26720428 PMC5432433

[11] Kaufmann C. R. , Dwyer K. M. , Crews J. D. , Dols S. J. , and Trask A. L. , Usefulness of Thrombelastography in Assessment of Trauma Patient Coagulation, Journal of Trauma and Acute Care Surgery. (1997) 42, no. 4, 716–722, 10.1097/00005373-199704000-00023, 2-s2.0-0030987123. 9137263

[12] Subramanian M. , Kaplan L. J. , and Cannon J. W. , Thromboelastography-Guided Resuscitation of the Trauma Patient, JAMA Surgery. (2019) 154, no. 12, 1152–1153, 10.1001/jamasurg.2019.3136, 2-s2.0-85073625174. 31596452

[13] Spahn D. R. , Bouillon B. , Cerny V et al., Management of Bleeding and Coagulopathy Following Major Trauma: an Updated European Guideline, Critical Care. (2013) 17, no. 2, 10.1186/cc12685, 2-s2.0-84876280594. PMC4056078 23601765

[14] Colman E. , Yin E. B. , Laine G. et al., Evaluation of a Heparin Monitoring Protocol for Extracorporeal Membrane Oxygenation and Review of the Literature, Journal of Thoracic Disease. (2019) 11, no. 8, 3325–3335, 10.21037/jtd.2019.08.44, 2-s2.0-85073332359. 31559035 PMC6753426

[15] Chlebowski M. M. , Baltagi S. , Carlson M. , Levy J. H. , and Spinella P. C. , Clinical Controversies in Anticoagulation Monitoring and Antithrombin Supplementation for ECMO, Critical Care. (2020) 24, no. 1, 10.1186/s13054-020-2726-9. PMC6971875 31959232

[16] Volod O. , Bunch C. M. , Zackariya N et al., Viscoelastic Hemostatic Assays: a Primer on Legacy and New Generation Devices, Journal of Clinical Medicine. (2022) 11, no. 3, 10.3390/jcm11030860. PMC8836477 35160311

[17] De Pietri L. , Bianchini M. , Montalti R et al., Thrombelastography-Guided Blood Product Use Before Invasive Procedures in Cirrhosis with Severe Coagulopathy: a Randomized, Controlled Trial, Hepatology. (2016) 63, no. 2, 566–573, 10.1002/hep.28148, 2-s2.0-84956754796. 26340411

[18] Rizvi G. , Marcinkowski B. , Srinivasa N et al., Impact on Blood Product Utilization with Thromboelastography Guided Resuscitation for Gastrointestinal Hemorrhage, Journal of Intensive Care Medicine. (2023) 38, no. 4, 368–374, 10.1177/08850666221126661. 36112899

[19] Kohli R. , Shingina A. , New S et al., Thromboelastography Parameters Are Associated with Cirrhosis Severity, Digestive Diseases and Sciences. (2019) 64, no. 9, 2661–2670, 10.1007/s10620-019-05597-4, 2-s2.0-85064086504. 30915655

[20] O′Leary J. G. , Greenberg C. S. , Patton H. M. , and Caldwell S. H. , AGA Clinical Practice Update: Coagulation in Cirrhosis, Gastroenterology. (2019) 157, no. 1, 34–43.e1, 10.1053/j.gastro.2019.03.070, 2-s2.0-85067182349. 30986390

[21] Wikkelsø A. , Wetterslev J. , Møller A. M. , and Afshari A. , Thromboelastography (TEG) or Thromboelastometry (ROTEM) to Monitor Haemostatic Treatment Versus Usual Care in Adults or Children with Bleeding, Cochrane Database of Systematic Reviews. (2016) 2016, no. 8, 10.1002/14651858.CD007871.pub3, 2-s2.0-84983347895. PMC6472507 27552162

[22] Nepal C. , Kc O. , Koirala M et al., A Retrospective Study Comparing the Effect of Conventional Coagulation Parameters Vs. Thromboelastography-Guided Blood Product Utilization in Patients with Major Gastrointestinal Bleeding, Journal of Clinical Medicine Research. (2023) 15, no. 11, 431–437, 10.14740/jocmr5022. 38189039 PMC10769601

[23] Walsh M. , Moore E. E. , Moore H. B et al., Whole Blood, Fixed Ratio, or Goal-Directed Blood Component Therapy for the Initial Resuscitation of Severely Hemorrhaging Trauma Patients: a Narrative Review, Journal of Clinical Medicine. (2021) 10, no. 2, 10.3390/jcm10020320. PMC7830337 33477257

[24] White N. J. , Ward K. R. , Pati S. , Strandenes G. , and Cap A. P. , Hemorrhagic Blood Failure: Oxygen Debt, Coagulopathy, and Endothelial Damage, Journal of Trauma and Acute Care Surgery. (2017) 82, no. 1, S41–S49, 10.1097/ta.0000000000001436, 2-s2.0-85015974964. 28328671 PMC5488798

